# The Role of Soil Fungi in K^+^ Plant Nutrition

**DOI:** 10.3390/ijms20133169

**Published:** 2019-06-28

**Authors:** Rosario Haro, Begoña Benito

**Affiliations:** 1 Centro de Biotecnología y Genómica de Plantas. Universidad Politécnica de Madrid (UPM) - Instituto Nacional de Investigación y Tecnología Agraria y Alimentaria (INIA), Campus Montegancedo UPM, Pozuelo de Alarcón, 28223-Madrid, Spain; 2 Departamento de Biotecnología-Biología Vegetal, Escuela Técnica Superior de Ingeniería Agronómica, Alimentaria y de Biosistemas, UPM, 28040-Madrid, Spain

**Keywords:** potassium, K^+^-solubilizers, K^+^ transporters, soil microorganisms, symbiosis with plants, mycorrhizal, endophytes

## Abstract

K^+^ is an essential cation and the most abundant in plant cells. After N, its corresponding element, K, is the nutrient required in the largest amounts by plants. Despite the numerous roles of K in crop production, improvements in the uptake and efficiency of use of K have not been major focuses in conventional or transgenic breeding studies in the past. In research on the mineral nutrition of plants in general, and K in particular, this nutrient has been shown to be essential to soil-dwelling-microorganisms (fungi, bacteria, protozoa, nematodes, etc.) that form mutualistic associations and that can influence the availability of mineral nutrients for plants. Therefore, this article aims to provide an overview of the role of soil microorganisms in supplying K^+^ to plants, considering both the potassium-solubilizing microorganisms and the potassium-facilitating microorganisms that are in close contact with the roots of plants. These microorganisms can influence the active transporter-mediated transfer of K^+^. Regarding the latter group of microorganisms, special focus is placed on the role of endophytic fungus. This review also includes a discussion on productivity through sustainable agriculture.

## 1. Potassium, Third amongst the Most Important Macronutrients for Crop Production

After nitrogen (N) and phosphorus (P), potassium (K) is one of the main nutrients required for plant growth, since it plays roles in osmotic adjustment and the regulation of cell membrane potential and pH. It also participates in the activation of enzymes involved in important metabolic processes, such as protein synthesis and photosynthesis [1]. While N and P are basic constituents of the main cellular macromolecules, K remains in soluble form in cells. K^+^ is the most abundant cation in the cytosol, where it is accumulated against a large transmembrane concentration gradient, maintaining an almost constant concentration of around 100 mM [2]. This high cytosolic K^+^ concentration represents approximately 2–5% of a plant´s total dry weight [3]. To maintain this physiological concentration, plants should ensure proper K^+^ uptake by the roots from the surrounding soil.

**Figure 1:**
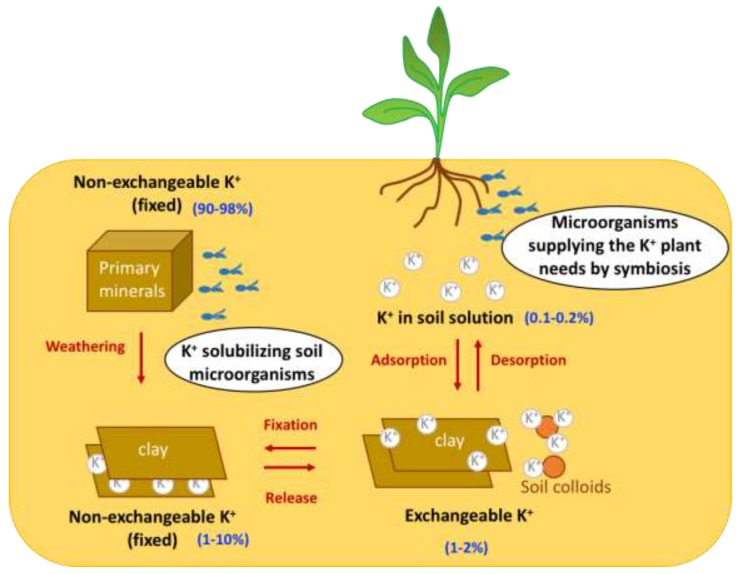
Role of microorganisms living in the soil in the dynamics of the K^+^ cycle. Scheme depicting the microbial strategies to improve K^+^ plant nutrition: K^+^ solubilizing microorganisms and microorganisms establishing symbiotic associations with plants.

Although potassium is the seventh most prominent element in the Earth's crust (preceded by silicon, aluminum, iron, calcium, magnesium, and sodium), it has low free availability for absorption by plants, and large areas of the agricultural land across the world are deficient in K. In fact, in the soil, K is found in four major pools with variable availability for plant absorption (Figure 1): (i) inside the crystalline structure of primary minerals (such as K-feldspars and micas), where it accounts for 90–98% of the total soil K content and is not available to plants; (ii) in non-exchangeable positions of secondary minerals, characterized by slow release, where K^+^ ions are adsorbed in the interlayer spacing of clay minerals, accounting for 1–10% of the total soil K content; (iii) in exchangeable forms characterized by rapid release which include K^+^ ions adsorbed by electrostatic forces that are released on clay and soil colloid surfaces, accounting for 1–2% of the total soil K content; and (iv) K in soil solution, which is readily available for immediate plant assimilation but accounts for just 0.1–0.2% of the total soil K content [4]. The last two forms of potassium are readily available for supply to plants, and are taken up in large quantities. 

The high concentration of K in healthy plants and the relevance to its cellular functions, as described above, means that K^+^ fertilization is an important agricultural practice for the optimization of crop production. Currently, strategies for soil K management rely on inorganic chemical-based fertilizers which, when abused, can cause serious threats to human health and the environment. To reduce the need to use chemical fertilizers to provide the nutritional needs of plants, microorganisms could be used as biofertilizers; however, although this approach is already used to supply N and P to plants, as yet, it has not been considered for K. Although it is known that the availability of K in soil depends more on cation exchange reactions than on the actions of microorganisms (Figure 1), the latter may also play a role in the bioavailability of K to plants and, thus, in the achievement of more sustainable agriculture.

## 2. Not Only Plant Roots Live Underground

From the plant physiologist´s point of view, soil in the natural environment provides plants with the mineral nutrients needed for their growth. However, the soil environment is a natural niche where numerous diverse microorganisms (e.g., fungi, bacteria, protozoa and nematodes) thrive along with the roots of plants. These organisms all obtain mineral nutrients from the soil to meet their physiological requirements. They are not evenly distributed in the soil but, rather, can be found in aggregates or “patches” at certain distances to plant roots or they can establish more intimate interactions in the proximity to the root surfaces of plants, a region known as the rhizosphere. Meanwhile, some of these microorganisms may colonize the roots, either inside the cells or in the apoplastic space between plant cells (named the endosphere) in such a way that, depending on their location in the soil, they may contribute differently to plant growth. In summary, there are three different mechanisms through which microorganisms can stimulate plant growth: (i) by altering the hormonal signaling of plants; (ii) by competing with the pathogenic microbial strains; and (iii) by facilitating the absorption of nutrients from the soil. In this review article, the third mechanism is explained in more detail.

Positive microbial interactions that promote plant growth by intervening in plant nutrition have been known for decades, including the nitrogen-fixing bacteria present in nodulated legumes and the arbuscular mycorrhizal fungi (AMF), which are the most recognized examples of beneficial root symbionts [5,6]. However, more recently, the focus has been placed not on individual species but on the knowledge of the bulk of the soil microorganisms that may affect, directly or indirectly, plant fitness and nutritional state. This has been identified in metagenomics studies as the “soil microbiome” [7]. The results of high-throughput sequencing studies from various soil samples have indicated that the rhizosphere is a hotspot of the microbial diversity with a huge variety of bacterial taxa [8], whose levels of abundance are probably determined though exudate production by the plants.

Unlike the bacterial microbiome, the fungal microbiome (or “mycobiome”) associated with plant roots has been poorly studied and characterized. This includes species co-existing in healthy plants, as well as those that exist in plants that thrive in different abiotic stressed environments. Amongst the large-scale projects that have attempted to characterize microbial life on the planet, the initial efforts of the Earth Microbiome Project (EMP, http://www.earthmicrobiome.org), have been focused on exclusively characterizing the bacterial microbiome, specifically its composition and impact on natural ecosystems. Therefore, the characterization of the mycobiome still needs to be carried out on a large scale, and its contribution to plant nutrition remains to be clarified. Meanwhile, several recent works have supported the importance of the mycobiota of the plant root for nutrition and adaptation to the environment of the host, especially the rhizosphere soil-dwelling filamentous fungi [9].

## 3. The Involvement of Soil Fungi in Plant Mineral Nutrition

Mycorrhizal fungi are terrestrial fungi that colonize plant roots. Amongst them, AMF are the oldest and most widespread fungi that establish mutualistic symbioses with plants [10]. In their obligate biotrophic interactions, AMF may provide their plant partners with up to 80% of the N and 100% of the P scavenged from the soil, both of which are required for plant growth and development, in exchange for up to 20% of the photosynthetically-fixed carbon produced by the plant [11]. Lipids may also be delivered to the fungus, which are needed for the production of fungal lipids [12]. Several transporters are involved in this trade between partners, which are emerging as key determinants in maintaining these functional symbiotic associations. With regard to K^+^, there is much less information about whether the microorganisms participate in the fluxes of this cation during the symbiosis, and if so, about the transporters involved. 

Another group of less known soil fungi that can live in close association to plant roots are the fungal endophytes. These fungi live inside plant tissues without causing symptoms of disease. They do not have an obligate biotrophic life stage and can live at least part of their life cycle away from the plant [13]. Different from the AMF, endophytic fungi do not form any typical or easily recognizable structures within the plant roots, but, based on recent works, it is now being considered possible that they can colonize most land plants [14]. These recent studies suggest that non-mycorrhizal interactions are more widespread than was previously thought, thereby opening the possibility that endophytes may also have relevant roles in plant nutrition [15]. The ability of endophytes to transfer mineral nutrients to plants is a relatively recent discovery, and the mechanisms of this transfer are still unknown in some cases. *Heteroconium chaetospira* is an example of a fungal endophyte that is capable of transferring N from decomposing organic matter to its host plant [16]. *Colletotrichum tofieldiae* [17] and *Serendipita indica* (previously named *Piriformospora indica*) [18] have demonstrated the ability to transfer P to their non-mycorrhizal host, *Arabidopsis thaliana*, promoting its growth under low-P conditions. Regarding K nutrition, *Serendipita* has recently been found to improve the K^+^ content in shoots of maize plants growing under salt stress [19].

The fact that the associations between endophytic fungi and plants are more frequent than was previously assumed, together with their ability to grow axenically in the absence of the host plant, are two practical advantages of the use of endophytic versus mycorrhizal fungi in biotechnological applications, such as biofertilizers. This makes it worthwhile to study endophytes and their contribution to the mineral nutrition of the plants.

## 4. Strategies of Soil Microorganisms for Improving K^+^ Nutrition of Plants

Soil-dwelling microorganisms associated with plant roots may improve K^+^ nutrition in plants via three different effects, especially when plants grow in lands affected by K^+^ deprivation. These comprise: (i) increasing the availability of K^+^ in the soil solution; (ii) mediating the K^+^ uptake from soil and then transferring it to the plant though intimate symbiosis interfaces; and (iii) inducing the expression of specific plant genes that encode for K^+^ transporters, which directly absorb the K^+^ from the soil or mobilize it from cellular organelles or plant tissues.

Below are some examples from the literature that correspond to each of the above strategies. It is worth noting that these strategies are not mutually exclusive, and one fungus can accomplish several strategies during its coexistence with the plant. This is the case for some ectomycorrhizas that are known to mobilize nutrients from rocks in addition to acquiring N and P from the soil and transferring them to the plant [20].

### 4.1. K^+^ Solubilizing Microorganisms (KSM)

Considering the high K content that plants accumulate (2–5% of the total dry weight), and that, for some crops, a large number of plants are unusable and are generally discarded, one obvious and sustainable strategy for K soil fertilization would be to use the crop plant residues to supply nutrients to other plants. In this case, the contribution of soil microorganisms to plant K^+^ nutrition would be carried out by cellulose degraders that would facilitate the release of cellular K^+^. 

Apart from microbial activities, the process of K^+^ solubilization from mineral rocks can be performed by rhizosphere microbes, which can extract K from soil particulates containing minerals enriched in immobilized K, such as orthoclase, illite, biotite, feldspar, and mica. Various rhizosphere microorganisms are reported to be K-solubilizers; these are mostly found in Asian countries in various crops [21]. The study of KSM has demonstrated the phylogenetic diversity of these species, including bacteria and fungi. Most of the isolates correspond to bacteria that belong to the phyla *Firmicutes (Bacillus* sp., e.g., *B. mucilaginosus, B. edaphicus*, etc.)*, Proteobacteria (Pseudomonas* sp.*,* or *Klebsiella* sp.), and *Actinobacteria (Arthobacter* sp.). Regarding the fungi species, most of them are included in the Ascomycota phylum, such as the filamentous fungi *Aspergillus fumigatus or A. niger* and the yeast *Torulaspora globosa* [21]. In addition, within the group of ectomycorrhizal fungi, some examples have been described as having the capacity to solubilize K from rocks and providing it to their respective symbiotic partners [22,23].

The mechanisms through which KSM can release K from K-bearing minerals in an accessible form for plant uptake and transport are unclear but appear to be mainly based on lowering the pH of the soil. These microorganisms basically excrete H^+^ or organic acids (such as citric acid, tartaric acid, and oxalic acid) into the soil, which results in the acidification of the surrounding environment of the microbial cells, promoting the solubilization of K [4,24].

An experimental study of the microbial capacity to solubilize K^+^ from minerals can be easily tested using the so-called Aleksandrov medium, which contains, among other components, potassium silicate and aluminum. The mineral solubilizing capacity of microbes develops with the appearance of a halo. Modifications to this medium through the addition of pH indicators dyes have been done to improve the visualization of microbial acid production and secretion [25].

Several studies have reported that inoculation with K solubilizing microorganisms exerts beneficial effects on the growth of different crop plants [4]. However, the use of K-solubilizing microbes as bioinoculants, needs further research in order for them to be considered as a realistic alternative for K^+^ mineral fertilization of agricultural soils.

### 4.2. Microorganisms Mediating the K^+^ Transfer to Plants by Symbiotic Interactions 

Increasing information about the roles of microbial transport proteins of the rhizosphere in N and P uptake from the soil, as well as the proteins that transfer them to the plants, is emerging. On the other hand, the number of plant transporters identified as responsible for the acquisition of these nutrients from the symbiotic interfaces is also increasing. However, this is not the case for K^+^. Although an improvement in the N and P content in plants colonized by mycorrhizal fungi has been clearly recognized [26], the effective involvement of symbiosis in plant K^+^ nutrition is still under study [27,28]. Several works have reported an improvement of the K^+^ content of the plants in symbiosis with mycorrhizal fungi [29,30,31,32]. However, until now, the mechanisms involved have not been uncovered in all cases. Interestingly, one study that was carried out with the ectomycorrhizal fungi *Hebeloma cylindrosporum* found a positive effect on plant growth under K^+^ limited conditions which was associated with the expression of a particular fungal transporter (HcTRK1, from *Hebeloma cylindrosporum* TRK1) [31]. This is an aspect that deserves more research, especially regarding the study of the K^+^ transporters of the fungal endophytes for which there is little available information. As a first attempt to advance this issue, the identification and functional characterization of the K^+^ transporters in the fungi and plants must be addressed. The identification of any microbial K^+^ transporter that mobilizes K^+^ to the plant would have a critical role in the K^+^ nutrition of plants. In the following paragraph, references to the possible K^+^ transporters involved in the interactions between fungi and plants are made.

### 4.3. Microbial Symbionts as Inducers of Plant Genes that Improve Plant K^+^ Nutrition

Besides the active role of soil microorganisms in providing the plant K^+^ supply, directly (by capturing K^+^ from the soil and transferring it to the plant) or indirectly (by solubilizing K^+^ in the soil and making it available for plant absorption), other beneficial effects of the symbionts on plant K^+^ fitness cannot be ruled out. For instance, changes in the gene expression of fungi and plants that regulate the levels of cellular signals or the production of phytohormones by the plant itself [33] or through synthesis by the associated fungus, may occur during symbiosis [34,35]. These cellular players may induce changes in expression that may ultimately activate the K^+^ transporters of the host plant itself, thereby improving its K^+^ content (Table 1). The next paragraph presents an example that seems to confirm this idea. 

## 5. The Dual Transportome for Plant K^+^ Nutrition of the Two Partners in Symbiosis

The soil microbiota and plant roots must take up K^+^ through transport systems from an external medium that can be extremely variable in composition and in which the K^+^ concentration can also be highly variable. Oligotrophic environments may have a low K^+^ concentration, and the soil organisms (microorganisms and plants) must express K^+^ transporters with a certain concentrative capacity to ensure that K^+^ reaches the appropriate cytosolic concentration. Alternatively, when the microorganisms live in symbiosis, they may be exposed to high levels of accumulated K^+^ within the plant tissues or in the cytosol. They must avoid high levels of K^+^ to ensure their physiological concentration of cellular K^+^ is maintained. In general, all soil inhabitants can grow well in either of these two K^+^ conditions, which indicates that they must have different K^+^ transport systems with different functions (inward or outward fluxes) and capacities. 

Despite the growing interest in determining the most relevant molecular players in soil K^+^ nutrition and in the K^+^ exchange between mycorrhizal fungi and plants, few data are available. This article presents some examples of interesting reviews on the fungal transportomes for mineral nutrients in mycorrhizal symbiosis. In these articles, the paragraphs referring to K^+^ transport are not very extensive [26,45,46,47]. 

### 5.1. The K^+^ Transporters of the Fungal Side

Most of the information currently available on the function of families of K^+^ transporters in fungi has been obtained in non-symbiotic fungi, mainly in *Saccharomyces cerevisiae* [48,49,50] but also in other non-conventional yeasts [51], such as the soil-dwelling *Schawnniomyces occidentalis*, [52], or in filamentous fungi such as *Neurospora crassa* [53]*, Ustilago maydis* [54] and *Magnaporthe grisea* [55].

**Figure 2:**
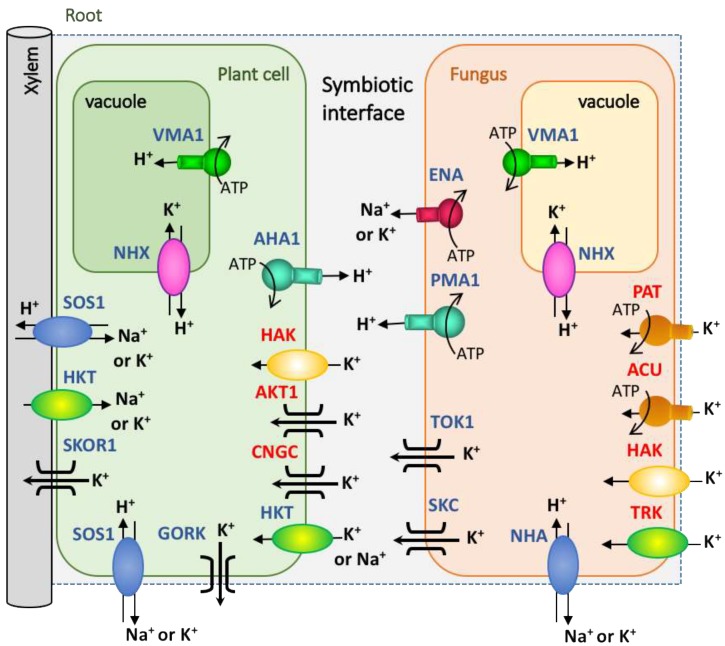
Proposal of the transportome for K^+^ nutrition in plant-fungal symbiosis. The different families of plant and fungal K^+^ transporters and channels identified so far are depicted. In red letters are marked the putative main players involved in K^+^ uptake. The H^+^-ATPases are represented as chief responsible for the maintenance of the membrane potential. In the fungal K^+^ acquisition, TRK, HAK, ACU or PAT would contribute taking up K^+^ at different ranges of substrate concentrations. Once inside the cells, K^+^ will distribute to fungal hyphae and could be accumulate in the vacuole or translocated out the cells. This K^+^ efflux systems could be mediated by channels (TOK, or SKC) or ENA-ATPases. The plant cells will take up K^+^ through high affinity (HAK transporters or AKT (Arabidopsis K^+^ Transporter) channels) or low affinity systems (e.g., CNGC (Cyclic Nucleotide Gated Channels)), depending of the available K^+^ concentration. K^+^ would be released in the xylem through SKOR-like (Stelar K Outward Rectifier) channels or any other systems for the distribution to shoots. To simplify the scheme, not all the transport systems of the vacuole have been included. In this figure HKT (High-affinity K^+^ Transporter), SOS (Salt Overly Sensitive), and NHA transporters are included because they may mediate K^+^ transport, although they have also been described as Na^+^ transporters. Other transporters (e.g., NHX), and channels (e.g., SKOR and GORK (Guard cell Outward Rectifying K)) could contribute to maintain the membrane potential and the ionic cellular homeostasis.

Amongst the fungal K^+^ transporters, there are two highly conserved families of proteins named TRK (derived from the Transporter of K^+^) and HAK (derived from High Affinity K) transporters [55], which are involved in K^+^ acquisition (Figure 2). TRK transporters are H^+^/K^+^ symporters, although they eventually can co-transport K^+^ with Na^+^ (sodium) [56] and have been proposed to function as a channel-like chloride efflux [57]. The first identification of a HAK transporter occurred in the soil fungus *Schwanniomyces occidentalis*, which showed a high concentrative capacity for K^+^ uptake at μM concentrations in the soil solution [52]. More recently, a new family of transporters named ACU ATPases (derived from Alkali Cation Uptake transporters) was also identified as being involved in K^+^ and/or Na^+^ uptake, showing a high affinity for these cations [54]. Finally, another type of K^+^ transporter is the PAT proteins (derived from P-type ATPase), which are somehow related to ACU proteins, because both are P-type ATPases, but are sufficiently phylogenetically distant to classify them in separate clusters [55]. Thus far, this ATPase has only been characterized in the aquatic chytridiomycete *Blastocladiella emersonii* [58,59], although homologous proteins have been identified in the genomes of other fungi [55]. It has been shown that fungi can accumulate K^+^ via one or more mechanisms of transport, and also, more than one representative may be present within a family of transporters. One example of this is *Hansenula polymorpha,* which has one TRK protein and one HAK protein, both with finely coordinated functions depending on the growth conditions [60]. A kinetic study of these transporters indicated that HAK1 shows a high affinity for K^+^, while the affinity of TRK1 is two orders of magnitude lower. On the other hand, TRK1 was not found to be transcriptionally regulated, while HAK1 was strongly induced in response to very low K^+^ concentrations and was down-regulated by the presence of K^+^. Similar behaviors were shown in the homologous systems of *Neurospora* [53,61].

Apart from the K^+^ uptake systems, there are other proteins in fungal membranes that mediate K^+^ efflux from the cells. Among others, the TOK (Tandem-pore Outward-rectifying K^+^) or SKC (ShaKer-like) channels, or the K^+^/Na^+^-H^+^ Antiporter named NHA and the group of P-type ATPases called ENA ATPases (derived from Exit NAtrium), may cover this function (Figure 2). The involvement of other K^+^ transporters or channels that currently remain non-identified in fungi may not be excluded.

Regarding the fungal symbionts, there is scant information on the functional characterization of K^+^ transporters in fungi associated with plants, and the information that exists is basically restricted to the characterization of one K^+^ uptake system and one K^+^ efflux system, called HcTrK1 and HcTOK1, respectively, which both belong to the ectomycorrhizal fungus *H. cylindrosporum* [56,62]. These authors showed that the fungus is involved in the K^+^ nutrition of the host plant *Pinus pinaster,* and they proposed that HcTRK1 is probably the K^+^ uptake system from the soil, while HcTOK1 mediates the K^+^ unloading from the fungal cells to the plant–fungal interface. 

### 5.2. The K^+^ Transporters of the Plant Side

The uptake and distribution of K^+^ in plants is carried out through a great variety of transporters and channels that are grouped into several families, some of which are common with fungi. Among the K^+^ transporters, the HAK family is one of the more numerous ones, and it includes HAK5, the main high affinity K^+^ transporter involved in the K^+^ uptake from the soil [63] (Figure 2). Other K^+^ transporters belong to the HKT family, which displays structural and sequence similarities with the fungal TRK transporters [64] and includes some members that are more involved in Na^+^ transport [65,66]. In addition, other plant transporters involved in K^+^ transport belong to the NHX (Na^+^-H^+^ eXchanger), CHX (Cation-H^+^ eXchanger), and KEA (K^+^ Exchange Antiporter) families, which are all cation-H^+^ antiporters. A few of them are expressed in the plasma membrane but most are located in the endomembrane system and in cellular organelles [67]. Regarding the families of K^+^ channels, there are the Tandem-Pore K- (TPK) and the Two-Pore Channels (TPC) [68]; the cyclic nucleotide-gated channels (CNGC) [69]; and the Shaker-like voltage-dependent channels, which include AKT1 that is involved in the entrance of K^+^ through the roots in Arabidopsis and in other plants [70]. Amongst this clutter of K^+^ transporters that are expressed in plants, what is clear thus far is that AKT1 and HAK5 are the chief players in K^+^ acquisition by the root epidermal and cortical cells [70] (Figure 2). The rest of the transporters participate in the K^+^ accumulation in vacuoles or in other organelles, or in the transfer to the shoot via the xylem for distribution to the whole plant.

Although there is much information on the main proteins involved in the K^+^ uptake from the soil and its regulation [71,72], little is known about whether any K^+^ transporter is preferentially expressed in the symbiotic interface and, therefore, may be involved in K^+^ exchange between the symbionts. Transcriptomic analysis may provide evidence of transporters whose expression may be relevant during symbiosis. In this sense, a review of the expression studies performed under these conditions is carried out in this work, and the results obtained are compiled in Table 1. As shown, currently, there are very few examples of plant K^+^ transporters that are induced or repressed during plant–fungal interactions. Such transporters belong to the group of HAK and CHX transporters and AKT-type inward K^+^ channels, which could be responsible for the K^+^ uptake to plant cells. Interestingly, a work has just been published in which a mycorrhiza-specific tomato K^+^ transporter, SlHAK10 (from *Solanum licopersicum* HAK10), which is exclusively expressed in cells containing arbuscules and seems to mediate the mycorrhizal K^+^ uptake pathway, is identified for the first time [39]. In addition, another protein induced during symbiosis is a channel type SKOR (Stellar K^+^ Outward-Rectifier), which, during the mutualist association, may be stimulating the K^+^ loading to the xylem. More efforts must be made to unravel the role of K^+^ nutrition in the beneficial interactions between fungi and plants, including the specific activities of every molecular players involved.

Summing up, there are many K^+^ transporters in plants and fungi that work through different mechanisms, some of which are shared between both organisms and others are more exclusive to one of the two partners. These transporters allow the absorption of the cation from the soil, its transport across cell membranes, and in the case of plants, across different tissues. However, currently, it is still unclear which of them are fulfilling the relevant functions of trading K^+^ in symbiosis. Figure 2 proposes the K^+^ transportome in plant–fungal interfaces including putative candidates that may be involved.

## 6. Inventory of K^+^ Transporters Candidates to Have a Role in Plant-Endophytic Fungal Symbiosis

As mentioned above, the study of K^+^ nutrition in the mycorrhizal symbiosis is in its infancy, and significant effort is being channeled towards overcoming the lack of knowledge on this issue. Undoubtedly, transcriptomic studies during symbiosis may provide interesting information on the transporters that are expressed, and, therefore, their probable involvement in K^+^ exchange and nutrition under these conditions. In parallel, and in the meantime, the genomic study of putative candidates can provide valuable and preliminary information in this regard. Here, we present a compilation of all the possible K^+^ transporters and channels existing in fungi that interact with plants with completely sequenced genomes. As an example, we studied the repertoire of K^+^ transporters of endophytic fungi using an in silico BLASTP search, which was performed against the endophytic fungi genomes sequenced by JGI (Joint Genome Institute) (https://genome.jgi.doe.gov/endophytic_fungi/endophytic_fungi.info.html).

At the time of this analysis, there were seventy-one endophytic fungi with completely sequenced genomes, and out of these, only fifteen genomes were available. The amino acid sequences of known K^+^ transporters of other fungi were used as queries (to identify TRK proteins, the TRK1 from *N. crassa*; for HAK proteins, the HAK1 from *N. crassa*; for ACU proteins, the ACU1 of *Ustilago maydis*; for TOK channels, TOK1 from *S. cerevisiae*; for SKC channels, *H. cylindrosporum* SKC1; for ENA proteins, ENA1 from *S. cerevisiae*; and, for PAT proteins, the PAT1 from *B. emersonii*). Homologous sequences were identified for all of them in all of the analyzed genomes (Table 2). The information in the table indicates that, as expected, there are many diverse K^+^ transport systems that could be functional during either the endophyte stage of fungal life or the free life stage in soil, or in both physiological situations. In all fifteen genomes analyzed, more than one type of putative transporter was found to exist as a candidate to mediate the K^+^ uptake. At least one TRK transporter (and maybe more than one) was present in all genomes and, additionally, most of them had at least one HAK (thirteen out of fifteen), or ACU protein (twelve out of fifteen). With respect to the putative K^+^ efflux systems, TOK and ENA ATPases existed in all fifteen genomes. Exceptionally, one PAT protein and one SKC channel were identified in the genome of only one fungus, *Periconia macrospinosa* or *S. indica,* respectively (Table 2).

It would now be interesting to unravel which of these transporters are expressed during symbiosis, provide K^+^ to the plant, and ensure sufficient K^+^ is present to allow proper growth of the fungus during symbiosis. With this information, biotechnological applications on the intervention of a specific transporter of endophytic fungi could be considered in the near future.

## 7. Conclusions: Soil Microorganisms for Fulfilling Sustainable Development Goals in Agriculture

Based on the guidelines issued by the FAO (Food and Agriculture Organization) to achieve the objectives of sustainable development in the near future, it is necessary to improve agricultural practices towards a sustainable system that improves the nutrition of crops while being respectful of the environment (Sustainable Development Goals (SDG): http://www.fao.org/sustainable-development-goals/en/). This initiative can be achieved by increasing the efficiency of mineral nutrient use to improve crop production while reducing the currently excessive use of mineral fertilizers, which may act as pollutants in nature. However, the last report of the FAO on world fertilizer trends and its outlook to 2020 indicates that the demand for mineral fertilizers is growing annually by 1.9%, where K is maintaining its ranking as the most demanded fertilizer [73].

The current information compiled here supports the importance of plant root microbiota for host nutrition and for adaptation to the environment. In this sense, soil microorganisms that co-exist with plant roots and influence plant nutrition can be exploited to diminish the use of mineral fertilizers. Although the role of fungi in plant nutrition has been studied in depth with regard to N and P, more effort is required to unravel the roles of soil-dwelling and endophytic fungi, in K nutrition. The efficiency of K use in agriculture could be effectively improved through, among others strategies, the inoculation of effective K solubilizing microorganisms from soils or the rhizosphere or endophytic fungi that transfer K to plants by means of specific transporters and/or channels. Biofertilizers such as nitrogen-fixing bacteria have been in use for a long time. P solubilizing bacteria and fungi have also been introduced to agriculture practices. However, the use of microorganisms that provide K to the plant, whether bacteria or more interestingly, mycorrhizal and endophytic fungi that live in close contact with plants, is still yet to be tested as an effective strategy.

## Figures and Tables

**Table 1:** K^+^ plant transporters regulated during the symbiosis with the fungi.

Family of Transporters	Transporter Name	Expression Level During Symbiosis	Plant Partner	Fungal Partner	Conditions of the Study	References
HAK	HAK (gene not specified)	Up	*Lotus japonicus*	*Gigaspora margarita*	Transcriptomic analysis during the symbiosis	[36]
	HAK (gene not specified)	Expressed	*Medicago truncatula*	*AM (Genus not specified)*	Transcriptomic analysis of transporters during symbiosis	[37]
	HAK17, HAK5	Expressed	*Pinus sylvestris*	*Cenococcum geophilum*	Transcriptomic analysis during symbiosis	[38]
	SlHAK10	Up	*Solanum lycopersicum*	*Rhizophagus irregularis*	qRT-PCR analysis in symbiosis in variable K^+^ conditions	[39]
K^+^ channels	AKT2, SKOR	Up	*Zea maydis*	*Claroideoglomus etunicatum, Septoglomus constrictum*	qRT-PCR analysis in symbiosis under salinity conditions	[40]
	SKOR	Up	*P. sylvestris*	*C. geophilum*	Transcriptomic analysis during symbiosis	[38]
	LbKT1, LbSKOR	Up	*Lycium barbarum*	*R. irregularis*	qRT-PCR analysis in symbiosis during drought stress and after K^+^ supply	[41]
	AtKAT1, AtKAT2	Up	*Arabidopsis thaliana*	*Serendipita indica*	qRT-PCR analysis in symbiosis under salinity conditions	[42]
CHX	Ortholog to AtCHX20	Up	*M. truncatula*	*R. irregularis*	Transcriptomic analysis during symbiosis under K^+^ deprivation	[43]
HKT*^1^	OsHKT1;5, OsHKT2;1	Up	*Oryza sativa*	*C. etunicatum*	qRT-PCR analysis in symbiosis under salinity conditions	[44]
	AtHKT1	Up	*A. thaliana*	*S. indica*	qRT-PCR analysis in symbiosis under salinity conditions	[42]

*^1^ HKT transporters can transport Na^+^ in addition to K^+^

**Table 2:** Families of putative K^+^ transporters identified in the genome of the endophytic fungi in JGI. (https://genome.jgi.doe.gov/endophytic_fungi/endophytic_fungi.info.html).

Fungi Name	Phylum*	Number of Genes Encoding Transporters/Channels Identified						
		K^+^-Uptake Systems				K^+^-Efflux Systems		
		*TRK*	*HAK*	*ACU*	*PAT*	*TOK*	*SKC*	*ENA*
*Ascocoryne sarcoides NRRL50072*	A	3	2	2	ND	2	ND	1
*Beauveria bassiana* *ARSEF 2860*	A	1	ND	2	ND	1	ND	5
*Cadophora sp.* *DSE1049 v1.0*	A	3	1	1	ND	3	ND	5
*Daldinia eschscholzii* *EC12 v1.0*	A	3	1	1	ND	1	ND	3
*Hypoxylon sp.* *CI-4A v1.0*	A	3	1	ND	ND	1	ND	2
*Hypoxylon sp.* *CO27-5 v1.0*	A	3	1	1	ND	1	ND	2
*Hypoxylon sp.* *EC38 v3.0*	A	3	1	1	ND	1	ND	2
*Microdochium bolleyi J235TASD1 v1.0*	A	5	2	2	ND	2	ND	5
*Paraconiothyrium sporulosum AP3s5-JAC2a v1.0*	A	4	1	2	ND	2	ND	2
*Periconia macrospinosa DSE2036 v1.0*	A	3	1	1	ND	3	ND	3
*Phialocephala scopiformis 5WS22E1 v1.0*	A	2	2	2	1	4	ND	1
*Piriformospora indica* *DSM 11827 from MPI*	B	2	1	ND	ND	1	1	2
*Rhodotorula graminis* *strain WP1 v1.1*	B	1	1	1	ND	2	ND	1
*Trichoderma gamsii* *T6085*	A	1	ND	2	ND	1	ND	2
*Xylona heveae* *TC161 v1.0*	A	2	1	ND	ND	2	ND	1

Phylum*: A, Ascomycota; B, Basidiomycota; ND, genes not detected in the genome
